# A compilation of experimental data on the mechanical properties and microstructural features of Ti-alloys

**DOI:** 10.1038/s41597-022-01283-9

**Published:** 2022-04-26

**Authors:** Camilo A. F. Salvador, Eloa L. Maia, Fernando H. Costa, Julian D. Escobar, João P. Oliveira

**Affiliations:** 1 COMARI Research & Innovations, Sorocaba, SP Brazil; 2 Department of Materials and Chemistry, Research Group Electrochemical and Surface Engineering, Vrije Universiteit Brussel, Brussels, Belgium; 3 Metallurgical and Materials Engineering Department, University of Sao Paulo, Sao Paulo, SP Brazil; 4 UNIDEMI, Department of Mechanical and Industrial Engineering, Universidade NOVA de Lisboa, Caparica, Portugal; 5 CENIMAT/I3N, Department of Materials Science, NOVA School of Science and Technology, Universidade NOVA de Lisboa, Caparica, Portugal

**Keywords:** Metals and alloys, Mechanical properties, Biomedical materials

## Abstract

The present work depicts a compilation of mechanical properties of 282 distinct multicomponent Ti-based alloys and their respective microstructural features. The dataset includes the chemical composition (in at.%), phase constituents, Young modulus, hardness, yield strength, ultimate strength, and elongation. Each entry is associated with a high-quality experimental work containing a complete description of the processing route and testing setup. Furthermore, we incorporated flags to the dataset indicating (a) the use of high-resolution techniques for microstructural analysis and (b) the observation of non-linear elastic responses during mechanical testing. Oxygen content and average grain size are presented whenever available. The selected features can help material scientists to adjust the data to their needs concerning materials selection and discovery. Most alloys in the dataset were produced via an ingot metallurgy route, followed by solubilization and water quench (≈58%), which is considered a standard condition for β-Ti alloys. The database is hosted and maintained up to date in an open platform. For completeness, a few graphical representations of the dataset are included.

## Abstract (summary)

**Table:** 

Measurement(s)	elastic modulus • mechanical strength (yield) • deformation at rupture • Vickers hardness • oxygen content • grain size • mechanical strength (ultimate)
Technology Type(s)	longitudinal and transversal pulse-echo ultrasound, nanoidentation, mechanical testing • mechanical testing (tensile), mechanical testing (compression) • Vickers hardness testing • inert gas fusion chemical analysis • linear intercept method
Sample Characteristic - Organism	metallic alloys
Sample Characteristic - Environment	room temperature

## Background & Summary

The number of peer-reviewed experimental investigations on the mechanical properties of Ti-alloys listed in the *Web of Science* exceeded four thousand five hundred (4500) in September 2021. An increasing trend in titanium research, observed over the last 30 years^1^, accompanies this aggregated result. Ti alloys have become essential structural materials to many industries, from aeronautical to biomedical. In the former case, their elevated strength-to-density ratio and thermal stability provide an optimal combination for the aircraft structure (skeleton) and engine parts (compressor)^2^. In the latter, Ti alloys are preferred candidates for bioimplants due to their low elastic modulus and superior biocompatibility, which excel most metallic systems^3^.

Nevertheless, even within this extensive body of research, finding a dataset that consolidates recent experimental data on Ti alloys containing multiple properties of interest has been challenging since new reports are published across a broad range of fields of study daily. While each area demands particular processing routes, testing methods, and analyses, data comparison becomes more intricate. Furthermore, different from other emerging areas in metallurgy, such as high-entropy alloys^4^, currently available titanium databases are either private^5^ or outdated^6^. We aim to bridge this gap by proposing a new open-source database focused on Ti alloys.

Typical experimental studies on Ti-based alloys can be divided into two groups. The first group presents analytic works, with a narrative centered on phase transformations. These works usually attain an in-depth microstructural analysis and do not report extensively on the mechanical properties^7,8^. On the opposite, the second group focuses on comparative analysis, which comprises a straightforward exploration of new compositions and their mechanical behavior^9,10^. These works tend to provide only simplistic microstructural analyses, primarily based on conventional (laboratory) X-ray diffraction. The present data article aims to establish common ground between these two initiatives. The studies compiled herein depict only well-balanced research^11–13^, with enough detail on both fronts (i.e., mechanical properties and microstructural features). Please check the Methods section for further information about how the papers were selected.

The mechanical properties of Ti alloys derive from the stability and the physical traits of their phase constituents. Traditionally, the design of Ti alloys is based on tailoring the stability of equilibrium phases such as α (HCP, low-temperature phase) and β (BCC, high-temperature phase), with the aid of different alloying elements^14^. Elements named β-stabilizers (Nb, Ta, Mo, V, Fe) reduce the transition temperature between α and β, known as β*-transus*, allowing the β-phase to show at temperatures lower than 882 °C. However, processing–structure-property relationships in Ti alloys are convoluted, as the β-phase can exhibit many solid-state phase transformations upon cooling. Thus, as a reference condition, Ti-alloys are usually water-quenched (WQ) from the β-phase field, hindering diffusion-controlled transformations and promoting the retention of the prior β-phase at room temperature (RT). Exceptions to this case involve the formation of martensitic phases, such as α’ (with hexagonal structure)^15^, α” (with orthorhombic structure)^16,17^, athermal ω-phase (also hexagonal)^18^, or active intermetallics^19^. The α” phase is often observed in shape memory alloys and is associated with double-yielding and non-linear elastic behavior. The presence of ω is of particular concern to several applications since it can severely impair the alloy ductility^20^.

As briefly introduced in this section, the Ti system is complex, so it is essential to gather data from previous works that report enough information on the composition, processing, microstructure, and mechanical properties. From a materials selection perspective, an open database might help with future comparative analyses, helping materials scientists to identify desired properties among known compositions. As for materials discovery, a comprehensive compilation of mechanical properties and microstructure data may allow researchers to spot unexplored regions of the vast available compositional space. Computational Materials Science works have achieved this in the past^21^; however, due to the lack of organized experimental data, modeling and predictions were primarily performed using theoretical data obtained from first-principles calculations^22^. The public availability of a routinely updated database will allow experimental scientists to act more proactively concerning alloy exploration^23^.

## Methods

### Data collection

At an initial stage, the authors selected 140 potential studies from the literature (WebOfScience, SCOPUS, ScienceDirect) based on a preliminary search using specific keywords (“titanium”; “alloys”; “ti alloys”; “mechanical properties”; “microstructure”; “experimental”). Full-texts were manually retrieved and organized, discarding studies that satisfied any exclusion criteria (next section). This dataset covers data from 1986 to 2021, with most studies being published after 2010 (approx. 84%).

We then extracted the properties of interest from each selected article and tabulated them in an online spreadsheet. Two authors worked independently at this step and registered relevant information in the comments section of each entry. In the case of studies exploring multiple compositions, each one was assigned to a single dataset record. WebPlotDigitizer^24^ was employed to extract data from graphics when needed. The three remaining authors received a random sample of 20% of the dataset for blind review as a final step. Authors only received articles they did not personally select on stage 1 for evaluation.

### Exclusion criteria

- absence of a compositional evaluation;
- absence of details on the processing route;
- no phase identification (minimal accepted: X-ray diffraction);
- less than two of the following mechanical properties reported: Young modulus, yield strength/ultimate strength/elongation, hardness.
- samples subjected to thermal treatments other than solution treated (ST) or stress-relief (i.e., aging, inter-critical tempering, complex heating/cooling cycles);
- for powder-metallurgy/sintered samples (f) - relative density lower than 90%.

### Blind review

Two independent reviewers received the entry’s digital object identifier (DOI) subjected to blind review. They were also provided with a list (Table 1) containing the mechanical and microstructural features to be extracted from the text (i.e., an empty table). The reviewers independently downloaded the full text and then obtained all the variables/parameters to the best of their ability. Later, the annotated values from the two reviewers were compared with each other. In case of divergences, reviewers reach a consensus in a debate with all authors. After settling on the most appropriate number/value for the variable in question, the observations field of that entry was filled with all pertinent details on how to obtain the data - e.g.“mechanical properties extracted from Table 5”; “Average grain size estimated based on Figure 7”.

**Table 1:** Properties and additional fields included in the database.

Property/Variable	Format	Unit	Description
id	Integer	—	Unique identifier of a material.
eid	String	—	A conventional material identification based on the composition.
formula	String	—	Pymatgen compatible formula (at.%).
R1	Integer	—	Unique identifier of a reference.
R2	String	—	DOI of a reference.
R3	String	—	Citation schema (e.g., surname2021).
F1	Binary	—	=1, the study reports the oxygen content.
F2	Binary	—	=1, the microstructural analysis employed a high-resolution technique.
F3	Binary	—	=1, the material shows a non-linear elastic behavior.
P0	Float	wppm	Measured oxygen content in weight parts per million (wppm).
P1	Float	μm	Estimated average grain size.
YM	Float	GPa	Experimental Young modulus.
YM_err*	Float	GPa	The error associated with the Young modulus.
YS	Float	MPa	Experimental yield strength.
YS_err*	Float	MPa	Error associated with the yield strength.
UTS	Float	MPa	Ultimate tensile strength or maximum compression strength (in compression tests).
UTS_err*	Float	MPa	The error associated with the ultimate tensile strength.
DAR	Float	%	Deformation at the rupture point or maximum reported strain (negative values for compression tests).
DAR_err*	Float	DAR_err	The error associated with the deformation at rupture.
HV	Float	HV	Experimental Vickers hardness.
HV_err*	Float	HV	The error associated with the hardness.
moe	Float	%	A classical parameter known as “molybdenum equivalent” that represents β-phase stability (see Eq. 1).
moe_class	String	—	A classification based on the β-phase stability. Possible values are: “rich”, “near”, “meta”, “stable” or “other”.
P1	String	—	Identification of the predominant phase (matrix).
P2	String	—	Identification of the secondary phase.
P3	String	—	Identification of the tertiary phase.
condition	String	—	Processing conditions to which the material was subjected: ST-WQ (solution treated and water quenched), ST-AC (ST and air-cooled), ST-FC (ST and furnace cooled), PM (powder-metallurgy), As-cast.
comments	String	—	Relevant information not included in other variables.
composition	String	—	Reported experimental composition, in at.%. Similar to the “formula” field, but enables direct integration with the *pymatgen.Composition* module.

*Errors associated with the mechanical properties are only listed if reported in the text, in tables, or measured directly from image error bars.

It is worth mentioning that blind review identified only 21 discrepancies in the dataset; 11 were approximation errors from measurements taken from images, and 10 were wrong or missing values (i.e., a value that was present in the study which was not compiled into the dataset by human error). Data readily available in text or table was always favored over data displayed in images only.

## Data Records

**Fig. 1:**
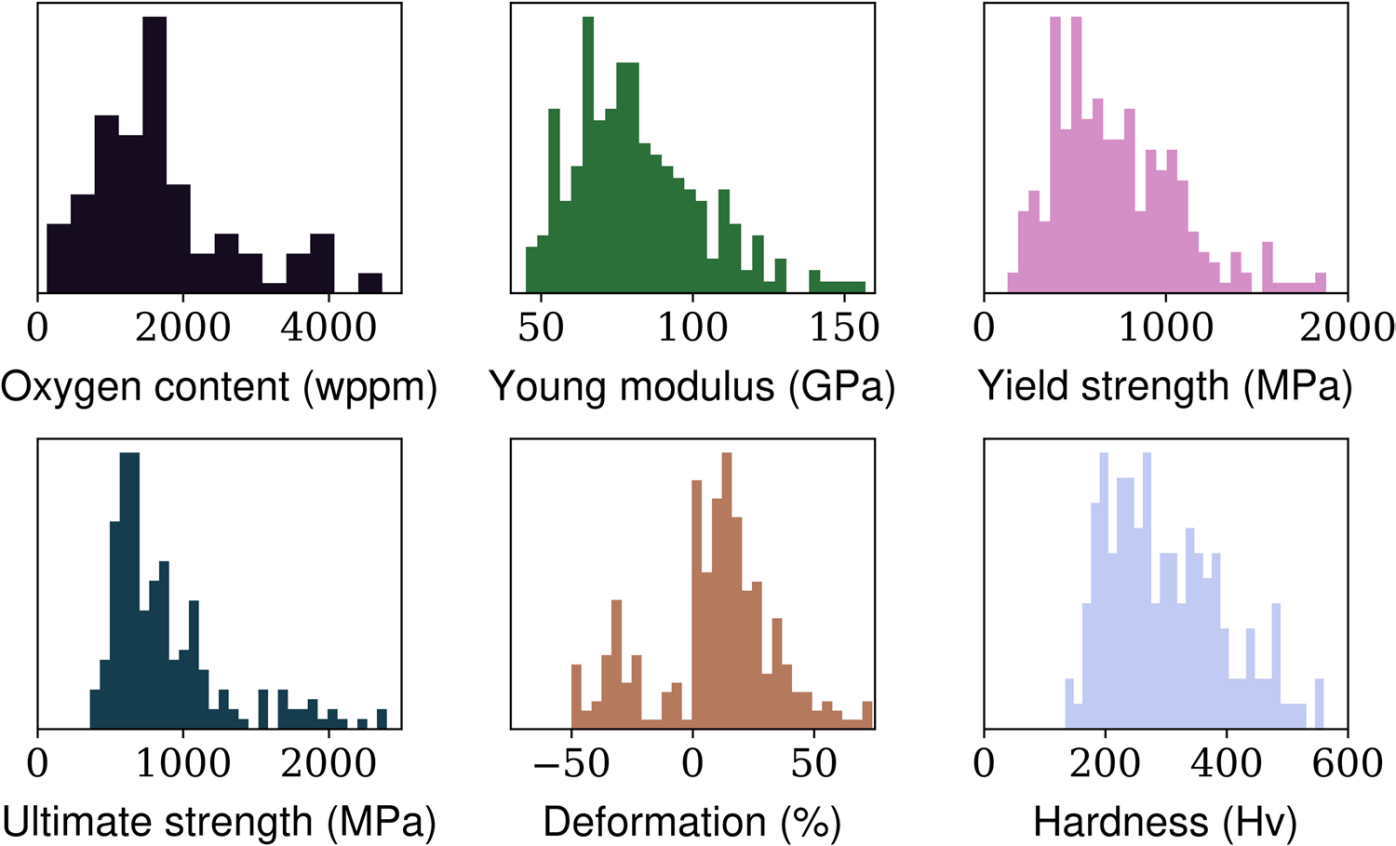
Frequency distributions of 6 essential variables from the dataset: oxygen content (in weight parts per million), Young modulus (GPa), Yield Strength (MPa), Ultimate strength (MPa), deformation at the rupture point (%), and Hardness (HV). The number of valid entries for each property is 121, 237, 271, 197, 257, and 152, respectively.

The dataset consists of 282 entries obtained from 105 high-quality experimental studies. These are surviving entries from 120 articles previously selected after manual curating, filtering, and blind review. Properties in Table 1 were compiled from the original texts to the best of the authors’ abilities to interpret the published results. Some properties in this dataset, such as the oxygen content (in wppm), the average grain size, and the elongation, are compiled in an identical format to recent works^4^. In this way, the elongation is considered positive in tensile tests and negative in compression tests. Unfortunately, not all critical properties are reported for all entries; this is especially problematic to address the impact of interstitial elements in Ti-alloys. Ti is highly reactive to oxygen, and minor variations in the oxygen content massively affect the phase transformations and mechanical properties^25^. Figure 1 depicts the frequency distribution of oxygen content (wppm) and the major mechanical properties contained in the dataset. The dataset is well balanced, including a broad range of strength, ductility, and hardness values.

A widely known parameter (included in this database) to represent the β-stability is the molybdenum equivalency (MoE). In simple terms, MoE is a weighted average of the composition (Eq. 1) that combines the critical concentrations of more than ten alloying elements into a Ti-Mo binary equivalent system. According to a recent review by Kolli & Devaraj^1^, MoE can also be used to categorize Ti-alloys into four distinct β-stability tiers: β-rich (0 ≤ MoE < 5), near-β (5 ≤ MoE < 10), β-metastable (10 ≤ MoE < 30), and β-stable (MoE > 30). In general, an MoE ≥ 10 is needed to retain the β-phase in a metastable condition after cooling from the β-phase field.

1 $$\begin{array}{c}{\rm{MoE}}=1.00{\rm{Mo}}+0.67{\rm{V}}+0.44{\rm{W}}+0.28{\rm{Nb}}+0.22{\rm{Ta}}+2.90{\rm{Fe}}\\ +1.60{\rm{Cr}}+1.25{\rm{Ni}}+1.70{\rm{Mn}}+1.70{\rm{Co}}-1.00{\rm{Al}}\end{array}$$

Data from this study can be found at Zenodo^26^ as a comma-separated-values (*.csv) file. The *.csv format is ubiquitous, and thus *.csv files can be easily imported into any software framework for further analysis. Moreover, the database can be easily updated using version control systems like git in this format.

## Technical Validation

### Outliers detection

Based on an in-depth analysis of key variables from the dataset (Fig. 1), we identified a dozen outliers, mainly regarding oxygen content and elongation. We double-checked these records and concluded that the oxygen contents in Xu *et al*.^27^ were indeed reported right; these specific studies are simply unusual, with relatively high oxygen additions^28^. Entries with extreme elongation were associated with specimens that did not fracture during mechanical testing; deformation at rupture values from these studies^25,29–32^ were set to a maximum of ±50%.

### Conflicting properties behavior

**Fig. 2:**
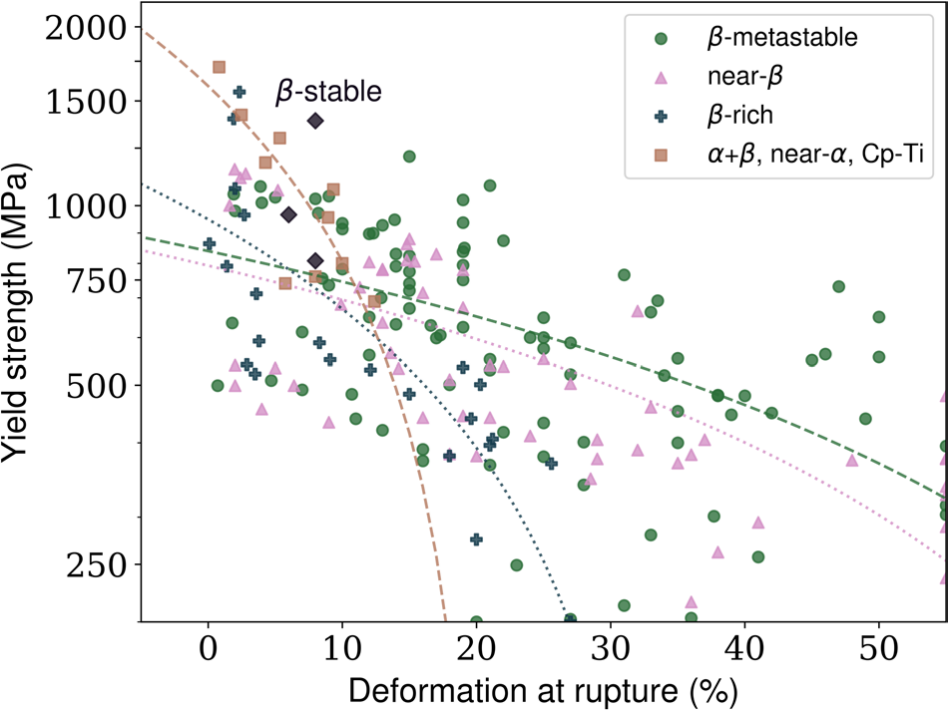
Using the dataset to create a yield strength (MPa) versus deformation at rupture (%) map. Entries are colored based on their MoE classification (see Eq. 1). The dashed and dotted lines are linear interpolations performed for each alloy class. The only three β-stable entries (MoE > 30) are depicted in purple (upper-left side).

A typical way of ranking structural materials is to observe strength-toughness relationships. Ideally, the material should present both features, but this rarely happens, as an increase in strength is often accompanied by a decrease in ductility^33^. Here we offer a yield strength versus elongation at failure map to depict such a relationship for the materials compiled in the present dataset (Fig. 2). Limit lines clearly illustrate the expected behavior of conflicting properties. Low-alloy compositions (near-α, α + β) present a high strength with relatively low ductility.

On the other hand, near β and β-metastable alloys display a broad range of strength and elongations. The present dataset shows an extended range for these properties and more data points compared with recent reviews. It is also interesting to see how the dataset can be easily used for materials design.

## Usage Notes

The dataset on Ti-based alloys is stored as a *.csv file that can be easily imported into any data analysis framework. Additionally, we provide a python script named utils.py (Python software foundation, version 3.8) with functions to filter the database, calculate the MoE parameter, and plot the figures from this article. We recommend the use of the *pymatgen.Composition*^34^ module to obtain pymatgen objects based on the formula for each data record. The use of pymatgen gives the user access to many other essential methods/functions inherited from pymatgen objects.

## Data Availability

The dataset and the utility script (utils.py) are available on Zenodo^26^, an open data repository. The python3 script is also available on GitLab (https://gitlab.com/comari/dax-ti), in which the users might obtain static (dax-ti-static) or a rolling release version (dax-ti-sid) of the project. The rolling release version will be continuously updated based on external requests. Researchers are encouraged to contribute to the database through GitLab or via e-mail, sharing their published data to expand the dataset.

## References

[1] Kolli R, Devaraj A (2018). A Review of Metastable Beta Titanium Alloys. Metals (Basel)..

[2] Huda Z, Edi P (2013). Materials selection in design of structures and engines of supersonic aircrafts: A review. Mater. Des..

[3] Zhang L, Chen L (2019). A Review on Biomedical Titanium Alloys: Recent Progress and Prospect. Adv. Eng. Mater..

[4] Borg CKH (2020). Expanded dataset of mechanical properties and observed phases of multi-principal element alloys. Sci. Data.

[5] Limited, G. D. Granta Design CES Edupack 2016. (2016).

[6] Gerhard Welsch, Rodney Boyer, E. W. C. *Materials Properties Handbook: Titanium Alloys*. (ASM International, 1993).

[7] Li T (2016). New insights into the phase transformations to isothermal ω and ω-assisted α in near β-Ti alloys. Acta Mater..

[8] Choudhuri D (2017). Coupled experimental and computational investigation of omega phase evolution in a high misfit titanium-vanadium alloy. Acta Mater..

[9] Matsumoto H, Watanabe S, Hanada S (2005). Beta TiNbSn alloys with low Young’s modulus and high strength. Mater. Trans..

[10] Majumdar P, Singh SB, Chakraborty M (2011). The role of heat treatment on microstructure and mechanical properties of Ti–13Zr–13Nb alloy for biomedical load bearing applications. J. Mech. Behav. Biomed. Mater..

[11] Zhao X, Niinomi M, Nakai M (2011). Relationship between various deformation-induced products and mechanical properties in metastable Ti-30Zr-Mo alloys for biomedical applications. J. Mech. Behav. Biomed. Mater..

[12] Obbard EG (2011). The effect of oxygen on α″ martensite and superelasticity in Ti–24Nb–4Zr–8Sn. Acta Mater..

[13] Zhang JL, Tasan CC, Lai MJ, Yan D, Raabe D (2017). Partial recrystallization of gum metal to achieve enhanced strength and ductility. Acta Mater..

[14] Banerjee D, Williams JC (2013). Perspectives on Titanium Science and Technology. Acta Mater..

[15] Semiatin SL, Seetharaman V, Weiss I (1997). The thermomechanical processing of alpha/beta titanium alloys. JOM.

[16] Kim HY, Ikehara Y, Kim JI, Hosoda H, Miyazaki S (2006). Martensitic transformation, shape memory effect and superelasticity of Ti–Nb binary alloys. Acta Mater..

[17] Hao YL, Li SJ, Sun SY, Yang R (2006). Effect of Zr and Sn on Young’s modulus and superelasticity of Ti–Nb-based alloys. Mater. Sci. Eng. A.

[18] Williams JC, Blackburn MJ (1969). Influence of misfit on morphology and stability of omega phase in titanium-transition metal alloys. Trans. Met. Soc..

[19] Devaraj A, Nag S, Muddle BC, Banerjee R (2011). Competing Martensitic, Bainitic, and Pearlitic Transformations in a Hypoeutectoid Ti-5Cu Alloy. Metall. Mater. Trans. A.

[20] Geetha M, Singh AK, Asokamani R, Gogia AK (2009). Ti based biomaterials, the ultimate choice for orthopaedic implants – A review. Prog. Mater. Sci..

[21] Salvador CAF, Zornio BF, Miranda CR (2020). Discovery of Low-Modulus Ti-Nb-Zr Alloys Based on Machine Learning and First-Principles Calculations. ACS Appl. Mater. Interfaces.

[22] de Jong M (2016). A Statistical Learning Framework for Materials Science: Application to Elastic Moduli of k-nary Inorganic Polycrystalline Compounds. Sci. Rep..

[23] Liu Y (2017). Materials discovery and design using machine learning. J. Mater..

[24] Rohatgi, A. WebPlotDigitizer. https://automeris.io/WebPlotDigitizer (2021).

[25] Umbelino dos Santos L, Campo KN, Caram R (2021). & Najar Lopes, É. S. Oxygen addition in biomedical Ti–Nb alloys with low Nb contents: Effect on the microstructure and mechanical properties. Mater. Sci. Eng. A.

[26] Salvador CAF, Maia EL, Costa FH, Escobar JD, Oliveira JP (2022). Zenodo.

[27] Xu Z (2020). Research on microstructure and properties of Ti-15Mo-3Al alloy with high oxygen content. Mater. Res. Express.

[28] Dehghan-Manshadi A, Kent D, StJohn D, Dargusch M (2020). Properties of Powder Metallurgy‐Fabricated Oxygen‐Containing Beta Ti–Nb–Mo–Sn–Fe Alloys for Biomedical Applications. Adv. Eng. Mater..

[29] Salvador CAFF (2017). Solute lean Ti-Nb-Fe alloys: An exploratory study. J. Mech. Behav. Biomed. Mater..

[30] Kuroda D, Niinomi M, Morinaga M, Kato Y, Yashiro T (1998). Design and mechanical properties of new β type titanium alloys for implant materials. Mater. Sci. Eng. A.

[31] Senkov ON, Scott JM, Senkova SV, Miracle DB, Woodward CF (2011). Microstructure and room temperature properties of a high-entropy TaNbHfZrTi alloy. J. Alloys Compd..

[32] Zhao GH (2017). New beta-type Ti-Fe-Sn-Nb alloys with superior mechanical strength. Mater. Sci. Eng. A.

[33] Ritchie RO (2011). The conflicts between strength and toughness. Nat. Mater..

[34] Ong SP (2013). Python Materials Genomics (pymatgen): A robust, open-source python library for materials analysis. Comput. Mater. Sci..

